# Norepinephrine: the next therapeutics frontier for Parkinson's disease

**DOI:** 10.1186/2047-9158-1-4

**Published:** 2012-01-13

**Authors:** Peter A LeWitt

**Affiliations:** 1 Department of Neurology, Henry Ford Hospital and Wayne State University School of Medicine, Detroit, Michigan USA

**Keywords:** Norepinephrine, Parkinson's disease, neuropharmacology

## Abstract

Tissue concentrations of norepinephrine (NE) are markedly decreased in various regions of the Parkinson's disease (PD) brain. As in the substantia nigra pars compacta, neuronal dropout and Lewy bodies are prominent changes affecting the locus coeruleus, which is the source of ascending NErgic projections. Despite the major roles of NE throughout the brain, there has been only minimal exploration of pharmacological intervention with NErgic neurotransmission. Cognitive operations, "freezing" of gait, tremor, dyskinesia, REM sleep regulation, and other aspects of brain function are tied into signaling by NE, and there is also evidence that it may have a role in the neurodegenerative process itself. This article reviews the reported pharmacological experience in PD therapeutics.

## Review

Successful treatment of Parkinson's disease (PD) with neurotransmitter replacement has placed dopamine "center stage" for understanding the pathophysiology of this disorder. The seminal research of Arvid Carlsson and other investigators in the 1950s elevated dopamine's role from that of a mere metabolic intermediate to the "star of the show" in PD and other brain disorders. Neglected by the attention given to dopamine, however, was the significance of another important CNS neurotransmitter, norepinephrine (NE). In nerve terminals containing the rate-limiting enzyme dopamine-β-hydroxylase, NE is formed in the next step in catecholamine synthesis beyond dopamine. Like dopamine, NE is involved in a wide range of cognitive, motor, and autonomic functions of the brain. Beyond its roles as a neurotransmitter, the actions of NE are involved in one or more mechanisms linked to neurodegeneration in the PD brain [1]. There has been only limited pharmacological experience exploring the clinical significance of modulating NE neurotransmission. This review will cover the highlights of this therapeutic research experience.

In PD, NE synthesis is greatly decreased throughout the brain. In several regions, NE content is reduced to less than half of its usual tissue concentration [2]. CSF concentration of dopamine-β-hydroxylase (the rate limiting enzyme for NE synthesis) is also diminished [3]. The loss of NErgic innervation is attributable to the prominent pathology found in the locus coeruleus (LC) [4]. From these paired brainstem structures, ascending projections arise that are distributed widely to the cerebral cortex and deeper structures [5]. Long before the significance of decreased dopaminergic innervation was understood as a key feature of PD, neuropathologists recognized that changes in the LC were as extensive as those affecting neurons in the substantia nigra pars compacta (SNpc) [6]. Beyond the dropout of NErgic neurons in the LC, the remaining pigmented neurons tend to be affected with Lewy bodies and Lewy neurites (similar to findings for dopaminergic neurons in the SNpc). Neuronal degeneration in LC precedes by several years the development of similar changes in the SNpc [7]. Although the cause(s) in PD for the progressive and relatively selective attack on both the LC and SNpc remains to be learned, neurons in both brain regions share in common an intracellular accumulation of neuromelanin pigment as well as the enzymatic apparatus for catecholamine synthesis and catabolism. These factors may confer vulnerability for neurodegeneration based on oxidative stress from metabolism of the neurotransmitters or their auto-oxidation [8].

Research into the etiology of PD has also given consideration to other ways that NE might be involved. One intriguing possibility comes from its influence on inflammatory mechanisms, which have been suspected to be involved in the common final pathway for the pathogenesis of PD (regardless of initiating cause) [9]. In animal experiments, NE inhibits gene expression leading to pro-inflammatory molecules (especially cytokines) originating in microglia, astroglia, and endothelial cells [1]. Other properties associated with NErgic innervation include the reduction of oxidative stress (by lessening the formation of nitric oxide and other intracellular reactive oxygen species), and lessening of both mitochondrial membrane depolarization and caspase activation [10]. As a result, the presence of NE innervation may protect against neurodegeneration in the SNpc dopaminergic neurons. Evidence for this possibility comes from experimental lesioning of the LC, which adds to the damage of dopaminergic neurons caused by the neurotoxin 1-methyl-4-phenyl-1,2,3,6-tetrahydropyridine (MPTP) [11-13]. In contrast, enhancing NE synthesis counters the toxicity of MPTP against dopamine-secreting neurons in experimental Parkinsonism [13,14]. Taken together, the experimental evidence suggests that a decline in NE synthesis might be a factor in the neurodegenerative disease process of PD.

As with dopamine receptors, adrenoceptors in the nervous system have a complexity that is conferred by both their localization and by their differing signal transduction properties. The effects of NE are governed by an intermingling of receptors with excitatory and inhibitory pre- and post-synpatic functions. NE acts through both through immediate neurotransmission and also by long-term potentiation (which facilitates synaptic plasticity). It also indirectly enhances glutamate release. The NErgic output from the LC has a number of physiological roles in the brain, including activation of cerebral cortex for functions such as vigilance, wakefulness, and circadian rhythms. NE input plays a role in monitoring of arousal and attentional systems, especially those with inherent novelty. Attention mechanisms are enhanced by the influence of NE in the prefrontal cortex, while short-term memory is facilitated by actions of this neurotransmitter in the hippocampus. Pertinent to neurological deficits in PD are additional roles of LC NErgic innervation with respect to autonomic functions and regulation of the sleep-wake cycle. NE afferents from the LC also influence firing patterns of SNpc neurons and release of striatal dopamine [15].

There has been only limited pharmacological research with CNS NE to achieve symptomatic therapy of PD. In several studies, drugs selectively interacting with NE have offered insight to roles that NE might play in treating motor, cognitive, and affective features of PD. For example, clinical manifestations of resting, postural, and action tremor in PD can be diminished approximately one-third by the β-adrenoceptor antagonist nadolol [16]. Another β-blocker, propranolol, was claimed to reduce levodopa-induced dyskinesias in PD patients (and without worsening Parkinsonism) [17]. Levodopa-induced dyskinesias have also been diminished by other interactions with NE neurotransmission. For example, treatment with the α2-adrenergic receptor antagonists idaxozan [18] and fipemazole [19] reduced involuntary movements without a reduction in the anti-Parkinsonian benefits of levodopa.

Other motor features of PD and its therapeutics have been investigated by means of a drug that can selectively enhance NE production in the brain. The L-*threo *form of dihydroxyphenylserine (L-DOPS, or droxidopa) is an unnatural amino acid developed for pharmaceutical purposes. Although lacking any pharmacological effects by itself, L-DOPS is capable of direct conversion to NE by L-aromatic amino acid decarboxylase (the same enzyme that converts levodopa to dopamine). L-DOPS has been extensively used to treat orthostatic hypotension by increasing peripheral NE synthesis [20]. If the decarboxylase inhibitors carbidopa or benserazide are co-administered with L-DOPS, then most of this NE precursor is blocked from metabolism outside of the CNS. Like levodopa, L-DOPS crosses the blood-brain barrier by a facilitated amino acid transport mechanism.

In Japan, where the drug has been marketed since 1991, L-DOPS underwent investigation to investigate the role of NE in advanced PD. In a multicenter placebo-controlled study, some patients experiencing freezing of gait and retropulsive imbalance exhibited marked clinical improvements from L-DOPS [21]. Following these studies, there have been no further randomized clinical trials, and so the question of L-DOPS effectiveness in the problems of advanced PD awaits further study. Even when L-DOPS is co-administered with a decarboxylase inhibitor, recent clinical experience indicates that this drug can improve symptomatic features of postural hypotension experienced by PD patients [20].

Beyond its effects on motor aspects of PD, the role of NE in cognitive functions has been of particular interest for pharmacological interventions. Since the LC provides extensive projections to frontal cortex, several investigators have hypothesized that modulating NErgic neurotransmission might help with the various types of cognitive deficits found in PD. In one small-scale study, administering a selective NErgic α-1 agonist was associated with improved performance for attentional deficits in nondemented PD patients [22]. Another investigation found that treatment with the α-2 adrenoceptor agonist clonidine (which acts to decrease NE release in the brain) led to improved spatial working performance [23]. The NE re-uptake inhibitor atomoxetine showed a trend for improving several frontal lobe measures of cognitive performance in a small open-label study of non-demented PD patients [24]. Apart from these few clinical investigations, the role of NE in the range of PD-associated cognitive impairments [25] has not been explored.

The increased risk for occurrence of depression affecting PD patients has been associated with neurochemical changes that include the LC projections to limbic structures [26]. As a result, the reduction in NE neurotransmission may offer a pharmacological target. This possibility was explored in a clinical trial testing whether reboxetine, a selective NE re-uptake inhibitor, could offer improvement for a major depressive episode in PD patients. With this drug, the outcome was quite striking, as shown by improvements in Hamilton Depression Scale scores that were comparable to the best that serotonin-selective reuptake inhibitors achieve in treating depression for non-PD populations [27]. During this clinical trial, no changes occurred in ratings of Parkinsonian severity.

The studies described above constitute the sum of investigations in PD with respect to NE and the various deficits associated with this disorder. Given the importance of NErgic innervation in the brain and its major loss in PD, it would seem that this neglected neurotransmitter deserves greater pharmacological attention. Dopaminergic therapy of PD has greatly changed the options for achieving a lasting control of PD disabilities. Despite this, there are many continuing challenges for the further development of PD therapeutics (Table 1). Some of these unmet needs may be related to the various roles of NE in the brain. The studies described above, however meager, suggest that augmenting or blocking NE neurotransmission has the potential to improve several motor and non-motor problems of PD. Furthermore, NE's possible role at protecting against the progression of PD awaits study. One opportunity to explore the role of NErgic functions in the brain and systemically is with the NE precursor L-DOPS, which offers a selective pharmacological probe for augmenting NE neurotransmission. Using L-DOPS, the author is planning to initiate clinical trials to study the effects of increasing brain NE for treating cognitive impairments and freezing of gait in PD patients.

**Table 1:** The unmet needs of Parkinson's disease therapeutics

• Progressive worsening of all clinical features	• Unpredictable immobility (freezing of gait, prolonged "off" states)
• Inadequate tremor control	• Autonomic dysfunction (postural hypotension, constipation, dysphagia)

• Balance impairment (particularly retropulsive imbalance)	• Sleep disturbance (fragmented sleep, rapid-eye-movement behavioral disorder)

• Postural disturbance (forward flexion)	• Depression, fatigue, and apathy

• Involuntary movements with dopaminergic therapy (dyskinesia, dystonia)	• Progressive cognitive decline (including impaired working memory and executive dysfunction even in the absence of dementia)

## Competing interests

The authors declare that they have no competing interests.
